# Rough is salient: a conserved vocal niche to hijack the brain’s salience system

**DOI:** 10.1098/rstb.2024.0020

**Published:** 2025-04-03

**Authors:** Luc H. Arnal, Noémi Gonçalves

**Affiliations:** ^1^ Université Paris Cité, Institut Pasteur, AP-HP, INSERM, CNRS, Fondation Pour l'Audition, Institut de l’Audition, IHU reConnect, Paris 75012, France

**Keywords:** nonlinear vocalizations, roughness, sensory salience, auditory communication, evolutionary significance

## Abstract

The propensity to communicate extreme emotional states and arousal through salient, non-referential vocalizations is ubiquitous among mammals and beyond. Screams, whether intended to warn conspecifics or deter aggressors, require a rapid increase of air influx through vocal folds to induce nonlinear distortions of the signal. These distortions contain salient, temporally patterned acoustic features in a restricted range of the audible spectrum. These features may have a biological significance, triggering fast behavioural responses in the receivers. We present converging neurophysiological and behavioural evidence from humans and animals supporting that the properties emerging from nonlinear vocal phenomena are ideally adapted to induce efficient sensory, emotional and behavioural responses. We argue that these fast temporal*—rough*—modulations are unlikely to be an epiphenomenon of vocal production but rather the result of selective evolutionary pressure on vocal warning signals to promote efficient communication. In this view, rough features may have been selected and conserved as an acoustic trait to recruit ancestral sensory salience pathways and elicit optimal reactions in the receiver. By exploring the impact of rough vocalizations at the receiver’s end, we review the perceptual, behavioural and neural factors that may have shaped these signals to evolve as powerful communication tools.

This article is part of the theme issue ‘Nonlinear phenomena in vertebrate vocalizations: mechanisms and communicative functions’.

## 1. An essential and universal vocalization

Vocal signals play an essential role in alerting conspecifics and promoting survival [1,2] by effectively conveying warnings, especially in environments where visibility is limited. One common strategy to intensify exogenous sensory perception and ensure powerful reactions in the listener is screaming. This loud vocal response to fear or distress stands out as one of the most potent vocalizations that one can produce—reflexively or voluntarily [3]—to trigger a maximal response in the receivers’ brain, as well as fast and efficient reactions [4,5]. While speaking and singing require input and training, screaming holds an immediate and innate position in our communications toolkit [6]: one shared by numerous species exhibiting comparable vocal properties and behavioural consequences [7–9].

The human newborn’s immediate and instinctual emission of a cry upon birth, devoid of prior rehearsal, not only clears airways but also serves as a fundamental communication signal [10]. The acoustic features of a baby’s cry can provide valuable information about their health state, making the newborn’s cry not just a sign of life but also an interesting diagnostic tool [11,12]. Being born at an immature stage, human neonates are totally dependent on the care of their parents and rely on their capacity to capture their attention using loud utterances such as screams [13]. Screams possess an inherent ability to grab attention and are very difficult to ignore. They carry ecologically relevant attributes and are notably salient, thereby standing to ensue rapid reaction, requisitioning attentional resources with priority over other inputs [4,5].

In humans, screams occupy a distinctive position within the vocal repertoire, rarely used in routine communication [4] and inappropriate for most adult social situations in Western cultures. Screams extract a high cost both from the speaker and the listener. In the speaker, they require high energy outputs, causing strain in the vocal folds as well as throat pain [14]. In the listener, they can evoke high arousal [15–17], emotional distress and anxiety [18]. Consequently, speakers typically scream only in moments of severe distress, using this vocalization sparingly to justify the associated costs to themselves and others in their social group. These physical and societal pressures arguably encourage the parsimonious use of such high-emergency signals.

Alarm vocalizations cover a range of vocalizations beyond screams, including various acoustic features (e.g. pitch and timbre) to convey affective [19] or semantic information [20]. Here, we mainly focus on primordial, non-referential vocalizations that are intended to capture attention and warn or deter aggressors [4]. In humans, we specifically define ‘screams’ as loud, salient and involuntary vocalizations that are typically characterized by vocal harshness. While the term ‘scream’ is scarcely used in other species, this definition intuitively applies to animal vocalizations emitted in response to highly urgent situations. This distinction separates screams from other vocalizations, such as cries, whines or roars. While cries, for instance, often signal distress or need, particularly in infants, alarm vocal signals like screams stand out for their intense urgency, aimed at alerting conspecifics to immediate danger rather than communicating a specific emotional state. Focusing on alarm-like screams sets them apart from other rough vocalizations—such as joyful or excited screams—that, while sharing acoustic roughness, serve different communicative purposes and often express positive arousal rather than distress [21]. Accordingly, this review centres exclusively on alarm and distress calls, excluding other emotionally expressive, non-alarm signals that do not function as urgent warnings. Given the instinctual and primordial nature of alarm vocal signals, we propose that these vocalizations could be regarded as a shared precursor of vocal signalling, suggesting that identifying inherently salient or arousing aspects of such inputs may enhance our comprehension of the evolution of perception and communication systems. We review the perceptual, behavioural and neural constraints that may have shaped these vocalizations to evolve into powerful tools for effective alarm communication. We argue that a deeper understanding of the perceptual, neural and behavioural mechanisms that underlie salience processing in humans and animals may illuminate the processes that have contributed to the preservation of these acoustic features in vocal alarm communication.

## 2. Vocal production

**Figure 1.**
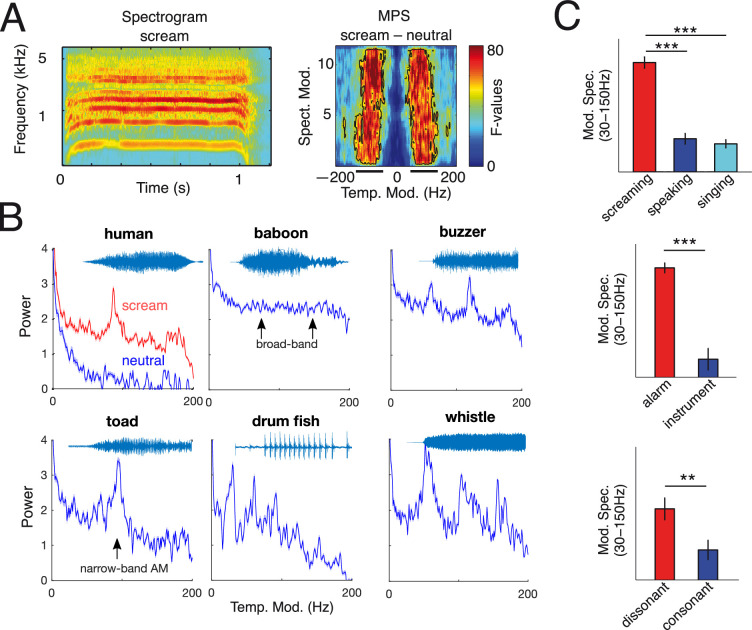
Acoustic properties of salient vocal signals across species. (A) Screamed vowel [a] spectrogram from one participant (top left); human scream vocalizations exploit a specific and isolated acoustic niche in the modulation power spectrum (MPS) (right) [4]. (B) Examples of temporal modulations in mammal (humans and baboons) screams, amphibian (toad) and fish (drum fish) vocalizations and artificial sounds (buzzer and whistle). L.H. Arnal, 2019, unpublished data. (C) MPS roughness. Top: screams (red), natural speech (dark blue) and musical vocalizations (a cappella) Centre: artificial alarm sounds versus musical instruments. Bottom: dissonant versus consonant sounds. Screams, compared with speaking and singing vocalizations, exhibit higher MPS values (e.g. average magnitude of temporal modulations in the roughness range (30−150 Hz). MPS values are also stronger in alarm and dissonant sounds compared with musical and consonant sounds, respectively. Roughness is also exploited in artificial alarm signals. ***p < 0.001, **p < 0.01, *p < 0.05. Error bars indicate the s.e.m. Spect. Mod.: spectral modulations, Temp. Mod.: temporal modulations. Adapted from Arnal *et al*. [4].

In mammals, vocalizations primarily result from the vibration of the vocal folds in the larynx [22], which are then filtered through the vocal tract [23]. While more typical vocalizations are driven by smooth harmonic changes in the larynx, more abrupt changes and tightening can cause chaotic dynamics leading to alert and distress calls, also referred to as nonlinear phenomena (NLP). The resulting sound possesses salient and arousing acoustic features efficiently acting upon sensory, emotional, cognitive and behavioural levels [24–26]. In mammals, NLP are more present in alarm and distress calls [27] and screams [4] than other vocalizations, serving as an effective tool to grab attention, to seek help from conspecifics [28] or to discourage aggressors [29] while preventing habituation [30]. The presence of NLP extends beyond adults; juveniles also produce similar vocalizations (non-humans [31–33], see also [34]; humans [35–37]), supporting the innate nature of such vocal behaviour in eliciting analogous responses [38]. While anatomical structures for vocal production can vary among animals, the use of nonlinear features for communicative purposes is widespread across the animal kingdom, extending beyond mammals (see, e.g. this special issue and following references on mammals: humans [4,39], marmots [40], also see [41], elephants [42], dogs [43,44], meerkats [45], manatees [46], chimpanzees [47], monkeys [48], koalas [49], giant pandas [50], altai pikas [51], sambars and muntjacs [52], wapitis [53], cows [54], red wolf [55], marine mammals: whales [56–58], dolphins [59], as well as birds such as sparrows [60], see also [61] and amphibians such as frogs [62]), suggesting an evolutionary continuum of warning communication across species (see examples in figure 1). Interestingly, cross-species studies have shown that mammalian species are capable of recognizing urgency in calls from other species, underlining the conservation of arousal cues in vocal communication. Humans have the capacity to accurately decode emotional perception of primate vocalizations [63–65], a capacity that even extends to non-mammalian vertebrates [66]. Even some reptiles, such as crocodiles, can react to vocal emotions conveyed by bonobo, chimpanzee and human alarm calls and are attracted to infant cries, although it is not clear whether this effect pertains to the detection of nonlinear cues [67]. Altogether, these observations suggest that despite diversity in vocal production mechanisms, many animals tend to utilize and produce similar acoustic features to be salient (see examples in figure 1B). The selection of nonlinear acoustic features for communication may be constrained by the receiving rather than the producing end, i.e. it may be optimized to align with the sensory processing constraints of the receivers.

## 3. Acoustic characterization

Vocal alarm signals have arguably emerged to maximize salience to ensure unconditional detection by the receiver. Therefore, analysing the acoustic structure of such sounds can reveal how natural communication has shaped vocal signals to maximize exogenous attention within the receivers’ sensory system. Among the numerous analytical methods used to measure NLP, the modulation power spectrum (MPS, see figure 1A, right panel) is particularly convenient in the current context—and more generally in auditory neuroscience—as it provides a neurally and ecologically relevant parametrization of sounds [68–71]. The MPS quantifies the power in temporal and spectral modulations by calculating the two-dimensional Fourier transform of a spectrogram (see figure 1A). NLP generally create distortions in the acoustic signal (e.g. chaotic modulations) that are visible on a spectrogram (subharmonics, chaos, frequency jumps) and can be quantified using the MPS. Focusing on human screams, we previously demonstrated that screams—compared to neutral vocalizations—enhance modulations in a specific acoustic/perceptual regime. This roughness range (see figure 1A, right panel) [4] reflects a restricted amplitude modulation (AM) frequency range [30−150 Hz] of the audible spectrum, perceived as salient and aversive, that has been preserved to communicate distress or danger. Interestingly, this special acoustic regime, ‘roughness’ appears to be almost exclusively used for alarm communication (figure 1B,C) but only minimally in neutral vocalizations such as speech or singing (figure 1C). Taken together, this supports the notion that screams—but not neutral vocalizations—occupy a specific acoustic niche in the audible spectrum—the roughness range—that is preserved to communicate high arousal (e.g. owing to danger or distress), emphasizing the biological and evolutionary relevance of such modulations. Many other animals (which generally have a more limited vocal repertoire than humans) appear to adopt this approach as well, exploiting rough cues to express urgency (see examples in figure 1B and above). Overall, sounds (whether natural or artificial) that aim to be salient often exhibit broadband spectral energy in the roughness range, sometimes combined with narrower-band amplitude modulations within the same frequency range (see figure 1B).

## 4. Roughness in culture

Auditory roughness was initially defined by Hermann von Helmholtz as the perception when two sounds that are tonally close are heard simultaneously [72], linking this attribute to musical dissonance [73,74]. Although initial characterization of roughness perception arose in psychoacoustic studies focusing on the comprehension of musical dissonance, a wealth of recent studies has supported the ecological and emotional relevance of this feature in our everyday lives. Roughness has been shown to be a critical aspect of music perception [75] as well as defining orchestral timbres [74]. Music composers often use non-harmonic distortions to influence our emotional responses [76,77]. Frightening scenes in films are often carried by ‘scream-like’ music (e.g. scratchy violins) exhibiting roughness and correlating with negative valence and higher arousal levels in spectators [17]. While it is tempting to suggest that the use of roughness is shared across cultures, its deployment may vary across cultures [78]. Existing research is largely limited to WEIRD populations, and further studies are needed to understand cross-cultural differences and universal aspects of roughness perception.

Beyond natural vocalizations, roughness is also exploited in sirens, fire alarms and medical alerts [4,79], suggesting that alarm sound designers empirically converge on choosing this attribute to enhance signals’ salience and to capture attention and/or convey danger. Amplitude modulations in the 30−150 Hz frequency range generate a rough sonic texture [80], inducing unpleasant or harsh subjective experience [76] commensurate with the power of such modulations [4]. Comparing the roughness of non-vocal sounds, we have shown that artificial alarms and dissonant intervals present stronger amplitude modulations in the roughness range than pitch-matched musical instruments and consonant sounds, respectively (figure 1*C*, middle and bottom panels). Beyond the auditory modality, roughness appears to be a fundamental sensory property that is used across different modalities [81]: strobe lights and tactile vibrators, which arguably have warning functions comparable to auditory buzzers—namely to capture attention—appear to exploit a very similar strategy to enhance perceptual salience by introducing fast temporal modulations in the sensory input [72].

## 5. Behavioural and affective reactions

**Figure 2.**
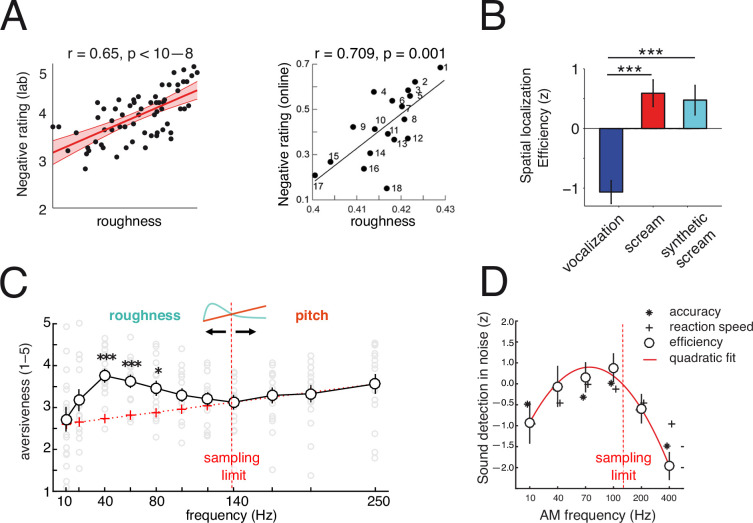
Affective reactions and behavioural impact of roughness. (A) Negative subjective rating increases with roughness assessed in lab conditions (right panel) and online [82]. Adapted from Zhao *et al*. [82]. (B) Roughness improves behavioural efficiency in spatially localizing rough vocalizations, whether produced naturally (screams) or artificially (synthetic screams), as compared with neutral, pitch-matched controls. ***p < 0.001, **p < 0.01, *p < 0.05. Error bars indicate the SEM. Adapted from Arnal *et al*. [4]. (C) Subjective assessment of temporal salience. Average subjective aversion (1–5 scale) follows a nonlinear profile. Above 130 Hz (i.e. the discretization limit), aversive response linearly reflects stimulus frequency (red line in inset). Below this limit, aversive responses are enhanced in the roughness range and are maximal at 40 Hz (nonlinear profile, green). Error bars indicate s.e.m. * and *** indicate significant *p*-values at 0.05 and 0.001. Adapted from Arnal *et al*. [5]. (D) Sound detection in noise. Participants’ performance (accuracy, reaction time and efficiency) follows an inverted U-shaped curve, optimally performing for amplitude-modulated sounds situated below the sampling limit in the roughness range. Efficiency is defined as the sum of *z*-scored accuracy and reaction speed. Quadratic fit (red parabolic curve) is calculated on efficiency data (L.H. Arnal, 2021, unpublished data).

Across artificial and natural sounds, roughness significantly impacts behavioural and emotional reactions. At the subjective level, the amount of roughness is highly predictive of participants’ aversion ratings to a sound, as shown in both lab and online experiments (see figure 2A, left and right panels [4]). Roughness also enhances behavioural efficiency (performance and speed) to spatially localize vocalizations: both natural and artificial rough screams are localized more efficiently than smooth neutral voices (figure 2B), demonstrating that roughness is a sufficient ingredient to enhance salience and facilitate behavioural reactions. In a psychoacoustic experiment [5], we tested the subjective impact of sounds (click trains of constant amplitude) on human participants across the roughness range and beyond (10–250 Hz). While one should expect to observe a linear profile of aversion commensurate with increasing sound intensity as a function of frequency (red line in figure 2C), sound aversion follows a nonlinear profile and is maximal in the roughness range, specifically at 40 Hz (figure 2C). Interestingly, the nonlinear increase in the aversion profile appears to be upper-bounded by the capacity of the brain to track fast temporal modulations and vanishes beyond the ‘temporal sampling limit’ (>130 Hz) at which temporal cues get fused into a continuous (pitch) percept. Beyond this limit, aversive responses linearly reflect the stimulus frequency (pitch). In addition to testing the effect of roughness on sound aversion and localization performance, we further hypothesized that such cues may enhance the detection of sounds presented at a low signal-to-noise ratio. This experiment showed that fast amplitude modulations in the roughness range (but not above) enhance the detection of tone carriers presented at low-level intensity (figure 2D, L.H. Arnal, 2021, unpublished data). Overall, the data converge to show that roughness can benefit the detection of very faint sounds, supporting that such acoustic features can extend the range of warning, even at a great distance between the emitter and the receiver.

Rough sounds not only have a considerable impact on perception but also on emotions. They are almost impossible to ignore or suppress perceptually, suggesting that their processing may be prioritized in a way that prevents habituation and competition with stimuli in other modalities. In extreme cases, overexposure to rough sounds like screams causes tremendous stress in the listener’s brain [83]. Persistent infant screams are often related to parental exhaustion, depression and even extreme responses sometimes leading to dramatic consequences such as shaken baby syndrome [84,85]. This also aligns with findings in rats showing that chronic exposure to screams has a considerable impact on cognitive and physiological processes, altering memory and monoamine levels, respectively [86,87].

In animals, studies show that during fear conditioning tasks, distress calls carrying rough amplitude modulations are emitted both in rats [88] and bats [89–91], arguably reflecting the animal’s state of fear and intent to deter or fly away from the experimenter. In chimpanzees’ social interactions, gaze direction is enhanced during screams compared with other vocalizations, suggesting that roughness may elicit arousal-mediated social engagement [92]. In the same way, parents spontaneously run to their screaming offspring and the AM tone’s roughness enhances mice’s attraction to a sound source as compared with sounds outside this range [93]. In humans, auditory roughness inhibits micro-saccades, highlighting the capacity of salient sounds to interrupt active visual exploration and rapidly orient attentional resources towards exogenous events [82].

## 6. Physiological responses

In parallel to evidence showing that rough sounds modulate subjective emotional responses, other works have investigated their influence on physiological and homeostatic body signals [94,95]. Several studies have linked stress and anxiety with cardiac changes in humans, with similar findings observed in animals [96–98]. In bats, Hechavarria *et al*. [89] used heart rate as an indicator of autonomic changes, showing that rough-like vocalizations accelerate heart rate potentially by stimulating neurons in the basal and central amygdala [99]. Whether this effect occurs via a direct routing from subcortical auditory primary regions to the amygdala [100] or indirectly through the auditory cortex [101] is still unclear (see below). In humans, baby screams often elicit greater heart rate reactivity responses, with an increase of skin conductance in parents, suggesting activation of the sympathetic nervous system [102]. Recent studies on hospital alarm systems, particularly in perioperative and critical care settings, suggest that these sounds may even impair patient recovery [79,103]. Whether this effect is attributable to the aversive features of these sounds needs clarification. Although rare in natural and artificial environments, rough sounds significantly affect human behavioural, perceptual and emotional responses, but in the absence of studies specifically focusing on this feature, its effect on human health remains to be clarified.

## 7. Salience and exogenous attention

Much of what we perceive as humans is shaped by top–down, endogenous attention, which serves to prioritize goal-oriented sensory sampling. However, certain stimuli ‘pop out’ and can trigger various reactions (emotional, reflex) even when outside the focus of attention. The way the auditory system prioritizes sensitivity to certain sound frequencies and acoustic features and ensuing reactions or emotions may differ across features. Salience is a key notion in understanding perception and ensuing behavioural reactions. This essential sensory attribute primarily determines whether a stimulus will be perceived or not and whether it may capture attentional resources ahead of other inputs. Although there is a long history of investigation into exogenous attention and salience in the visual modality [104–106] and of emotional responses to vocalizations in the auditory modality [107–112]—both primarily in humans—whether specific physical features (beyond stimulus intensity) can make stimuli more or less salient in other modalities is unclear. The neural mechanisms underlying attentional capture by rough features—whether acoustic or tactile—remain understudied. Furthermore, roughness is unlikely to be the only perceptual consequence of overblown production systems, and future research should explore additional co-varying features that may contribute to perceptual salience in a similar or complementary manner.

**Figure 3.**
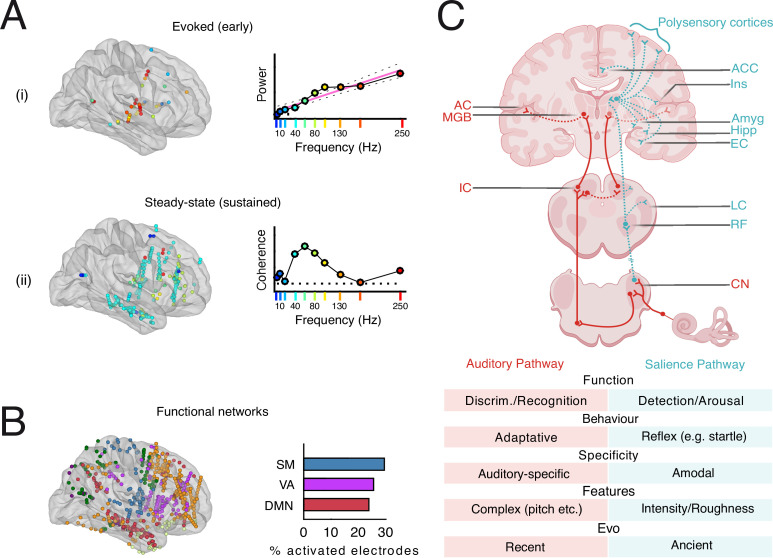
Dual pathways hypothesis. (A) The nonlinear patterns of subjective aversion (see figure 2) are best explained by the combination of (i) the linear recruitment of the classical auditory system as a function of click train frequency (top panels) and (ii) the nonlinear, sustained (steady-state) entrainment of neural responses in a widespread network of limbic and subcortical brain regions (bottom panels). (B) Functional networks analyses that show rough frequencies preferentially entrain neural activity in intracranial electrodes belonging to the sensorimotor network (SM), the ventral attention/salience network (VA/SN) and the default mode network (DMN), but not the dorsal attention/fronto-parietal network. Adapted from Arnal *et al*. [5]. (C) Dual neural pathways and related functions of the classical auditory system (red) and the salience system (blue). According to our hypothesis, the neural patterns generated by rough sounds are best compatible with the recruitment of a non-canonical, reticular pathway. This pathway arises in the dorsal cochlear nucleus (CN) and relays fast temporal acoustic patterns to various subcortical and cortical regions of the SN via a series of diffuse nuclei involving the reticular formation (RF). We suggest that non-canonical pathways play a crucial role in activating arousal and triggering fast responses to abrupt changes (acoustic transients) and can be probed using temporally salient, rough sounds. Table: the dual pathway hypothesis makes a series of predictions with regard to the respective functions and features of the two sensory systems. ACC, anterior cingulate cortex; Ins, insula; AC, auditory cortex; MGB, medial geniculate body; Amyg, amygdala; Hipp, hippocampus; EC, entorhinal cortex; IC,inferior colliculus; LC, locus coeruleus; Discrim., discrimination.

In the following, we focus our review on recent evidence suggesting that rough sounds target and efficiently impact the cortical brain network involved in exogenous attention, the *Salience Network* (SN). We hypothesize that these circuits emerge from specific neural *salience pathways* that constitute the entry point to a general arousal system, the *salience system* (figure 3C).

Current theories posit two distinct—endogenous and exogenous—attention systems [113] that interact to guide behaviour [114]. Functional connectivity analyses of magnetic resonance imaging (fMRI) data corroborate this distinction by isolating two distinct networks in the human brain: a ventral attention SN that corresponds to exogenous attention and connects anterior cingulate and insular cortices with subcortical and limbic structures, and a dorsal endogenous attention, fronto-parietal network (FPN) linking frontal and parietal cortical regions [115]. Activity in these networks relates to distinct functions: while the FPN affects executive-task performance, the SN plays a role in monitoring relevant exogenous events. Interestingly, the SN indexes individuals’ anxiety and is similarly activated by aversiveness or pain [116], supporting the view of a general, amodal *salience system* involved in modulating the perceiver’s arousal [117,118] in a bottom-up, driven manner. This *salience system* may presumably be shared by vertebrates [116] and may constitute an ancestral means for initiating behavioural and arousal responses (see also [117,118]).

Whether the postulated salience system is situated downstream of canonical sensory systems or whether another *salience pathway*—bypassing the more contextual processes elaborated at the cortical level—directly activates the salience system remains uncertain [119–121]. While the existence of direct pathways has been postulated and tested in the visual [122] and auditory [108] modalities, evidence for a non-canonical pathway to the salience system remained scarce (or marginal) until recently. In the following, we will review a series of studies from animal and human experiments suggesting that neural responses to salient, rough sounds are hardly compatible with the sole recruitment of the classical auditory system. Instead, these neural patterns may suggest *a dual pathway model* in which these sounds can additionally recruit ancillary, amodal pathways particularly sensitive to the temporal features of rough sounds. In turn, given the ancestral nature of these arousal/salience systems, we propose that the preservation of this acoustic niche for alarm signalling across species may reflect the conservation of these ancient and vital circuits throughout evolution (see figure 3C).

## 8. Neural responses to rough sounds

Neuroimaging studies in humans have shown that rough, unpleasant sounds not only activate the auditory cortices but also the amygdala, an area involved in fear and danger processing that shares reciprocal connections with the auditory cortex [123]. A reverse correlation analysis shows that rough features efficiently activate the amygdala regardless of the context, whether vocal, artificial or music [4]. Beyond the amygdala, other regions such as the anterior insula area and the anterior cingulate cortex display greater fMRI blood oxygen level-dependent signal (i.e. higher oxygen consumption reflecting an increase in brain activity) during the perception of crying infants [83]. Unpleasant, rough sounds (nails on a chalkboard, metal scraping, dentist drill, scratchy violin) can recruit a widespread network of brain regions, including limbic and paralimbic areas such as the amygdala, the nucleus accumbens (which can mediate reward or aversion processing [124]), the insula (involved in affective/emotional processing and autonomic functions [125]), the putamen (learning, reward, cognitive processing [126]), the thalamus and the cerebellum [5,127]. Consistent with Helmholtz’s original definition of roughness, dissonant sounds elicit unpleasantness, possibly via the recruitment of paralimbic and neocortical regions, including the para-hippocampus and precuneus (involved in memory, sensory processing, attention) [128]. Interestingly, human screams also have privileged access during sleep and increase slow theta waves and spindles [129], suggesting that roughness, as featured in alarm clocks and buzzers, may target sleep-related arousal systems. By analogy with the assumption that visual stimuli can impact subcortical regions such as the amygdala through direct subcortical pathways, rough sounds may be directly routed to subcortical and limbic regions involved in aversive emotional reactions, pre-empting the more detailed analyses performed by higher-level cortical networks.

## 9. Recruitment of a salience system

While the auditory system is the principal and decisive recipient of acoustic stimulation, the potential role of low-level or ancillary emotional pathways needs to be investigated more thoroughly. By measuring neural responses to click trains of varying rates during intracranial recordings in humans, we gathered evidence that temporally salient sounds synchronize a widespread network of subcortical nuclei (amygdala and hippocampus) and cortico-limbic regions that belong to the ventral attention SN (figure 3B). More specifically, acoustic temporal modulations between 30 and 80 Hz induce nonlinear enhancements in affective responses (subjective aversion) commensurate with increased neural synchronization. The neural pattern spans a network of temporal lobe areas, including the hippocampus, amygdala and insula, all particularly relevant to salience and affective processing. Revealing consistent nonlinear effects of sound roughness at the neural, affective and behavioural levels, this work consistently exposed a clear dichotomy in auditory and salience networks in aversive responses to this feature (see figure 3C and [5]). These findings have been replicated in several recent studies corroborating that steady-state entrainment in the roughness range (and particularly at 40 Hz) is not restricted to classical auditory regions but recruits a much larger network of medial brain regions [5,130–132]. Altogether, the widespread and sustained neural patterns in response to rough sounds evidenced in intracranial studies are incompatible with the sole recruitment of the classical auditory system. These observations, departing from typical routing and processing schemes in classical auditory pathways, suggest that these sounds additionally entrain neural activity in a *salience system* via non-canonical pathways leading to medial cortical and limbic regions [5,130] (figure 3C).

Interestingly, numerous studies in the literature demonstrate that responses to sounds in the 40 Hz range are affected in various clinical conditions (e.g. anxiety, Alzheimer’s disease, schizophrenia [133]) or by pharmacological manipulations, e.g. [134]. Of note, these diseases are rarely considered as selectively altering auditory functions but often present with less specific impairments involving attention, sleep or wakefulness—that can, but do not necessarily involve audition. Our hypothesis raises the intriguing possibility that impaired entrainment at 40 Hz in these conditions might involve defects in the salience instead of the auditory system.

## 10. Non-classical pathways: the dual pathway hypothesis

In the auditory domain, loud sounds induce arousing startle responses that have been known and extensively studied for a long time in humans and animals [135,136]. Recent studies in rodents showed that, beyond the fast generation of the startle motor reflex, such sounds further propagate via a non-primary auditory, ‘reticular-limbic’ system into deep brain regions, including the forebrain [137] and entorhinal cortex [138] (see also table figure 3C). This pattern of projections may account for the propensity of these sounds to ignite sympathetic physiological responses. Interestingly, neurons along this pathway can respond to broadband noise (but not to pure tones) at softer intensities than are required to elicit a motor startle reflex, thereby ensuring the efficient transmission of relevant signals to brain regions involved in fast (not necessarily reflex), adaptive reactions and learning [138]. This model is also supported by a series of seminal works performed in animal models in the 1960s, showing that brain and behavioural responses to sounds can be maintained despite the physical destruction of canonical auditory connections in the inferior colliculus [139]. Although more work is needed to clarify the exact pathways, it is possible that this non-canonical system corresponds to the ascending reticular activating system (ARAS), which is also characterized in human studies [140], although the terms ‘non-specific’ or ‘extralemniscal’ [141–143] may also refer to a similar notion and system. The ARAS system is principally well known for its role in sleep regulation and arousal activation [144], evoking waking from sleep in response to sensory stimuli and enhancing arousal in response to salient stimuli (see table figure 3C and [145]). This system is considered an evolutionarily ancestral and general arousal system possibly involving neuronal reticular pathways that have been hypothesized to evolve from medullary cells of teleost fish [117,118]. Until recently, we lacked both experimental evidence and linking hypotheses to support the view that this system plays a foremost role in perception and attention. However, adding to the discovery of the SN in humans [146,147], a series of very recent studies in animal models have revealed that the ARAS projects to various limbic regions and dynamically regulates the SN [148]. These crucial observations provide timely anatomical and functional evidence in favour of a salience pathway originating in the ARAS and ultimately projecting to (and exogenously controlling) the SN.

While recent research has elucidated the perceptual and neural processing of roughness in humans, there is a notable lack of comparable studies in nonhuman animals. Whether these sounds are perceived and processed by the proposed salience system in other species remains speculative at this point. Future comparative research should aim to systematically relate the use of roughness in alarm vocal signals to their behavioural, affective and neural effects across a broader range of species, e.g. from mammals to reptiles, to better understand the evolutionary and cross-species relevance of these features for alarm communication.

## 11. Conclusions

Several neural mechanisms may account for the propensity of rough and nonlinear vocalizations to enhance arousal in the receiver. Stemming from the observation that nonlinear vocal phenomena in mammals exhibit chaotic features, several authors have argued that the resulting sounds enhance attentional capture and prevent habituation owing to their unpredictability [8,40]. While this hypothesis is interesting and possibly correct, the evidence collected in this review points to another—although compatible—sensory phenomenon, pertaining to the capacity of these rapid temporal features, such as repetitions of acoustic transients or amplitude modulations, to entrain neural responses in a sustained manner over time. In this view, temporal salience, akin to a strobe light or tactile vibration effect, arises from the bombardment of sensory systems just below their sampling (fusion) limit, resulting in widespread synchronization throughout regions involved in salience processing. According to the dual pathways hypothesis, transient acoustic stimuli are not only carried by the auditory system but also via an evolutionarily ancestral, amodal salience system that is responsible for modulating the receiver’s arousal state (figure 3C). That fast repetitive features can be found in the communication signals of a wide array of vertebrate species, such as fishes and toads (figure 1), suggests that they may have been conserved over the course of evolution, which could date back to early adaptations of primordial sensory pathways [7]. This view contrasts with less urgent, more complex*—*e.g. tonal or harmonic—signals that arguably arose later to populate other acoustic niches of the audible spectrum and require more advanced processing involving auditory cortical regions.

In our hypothesis, the salience system is specifically sensitive to a restricted range of stimulation rates and may be probed using temporally salient sounds. If this system is mostly sensitive to impulses, by repeating impulses in time at various frequencies, it should be possible to identify the preferred resonance rate of this system. Therefore, temporally ‘enriching’ stimuli up to the sampling limit of the salience system might enhance stimulus detectability and increase subjective salience. Beyond this limit, events are not perceived as discrete but rather as continuous (pitch) and may not be perceived as salient any more. Altogether, these findings point to a supramodal neural correlate of temporal salience: the aversive sensation induced by rough sounds results from the persistent, exogenous synchronization of large-scale networks involved in salience—rather than specifically auditory—processing. This also suggests that the negative perception induced by rough sounds such as dissonant intervals [149], alarm sounds [4] or annoying vocal effects (e.g. vocal fry [150]) might result from their capacity to exogenously hijack brain networks involved in salience, aversion or pain processing [116]. In addition to the large extent of spatial synchronization patterns, it is remarkable that these networks preferentially resonate in a frequency range (30–150 Hz) that matches a well-known endogenous brain rhythm, the so-called gamma band [151]. Neuronal synchronization in the gamma range has been proposed as a mechanism to subserve the selective routing of bottom-up information in the brain [152,153]. Consequently, stimulus-driven, privileged entrainment in the gamma range by rough sounds may reflect the exogenous recruitment of attentional and arousal-related brain regions to ultimately enhance perceptual salience. Another interesting feature of this phenomenon is that, unlike early evoked auditory responses, steady-state sustained electrophysiological responses in the gamma range do not seem to habituate: their magnitude remains stable even after several repetitions of a sound (L.H. Arnal, 2021, unpublished result). This aspect is particularly relevant in the context of vocal communication because preventing neural habituation may ensure that the processing of alarm sounds is always prioritized over other environmental events.

This theoretical framework also has interesting implications for the understanding of salience in acoustic communication and supports the hypothesis that temporally enriching sounds in time—in the roughness range—amplify sensory salience and improve neural and behavioural efficiency. This nicely accounts for the observed convergence of roughness as a privileged acoustic niche to warn conspecifics [4]. Showing that such sounds recruit salience systems in the human brain and enhance perception, we confirm the fitness of these sounds to ultimately promote the efficient transmission of signals that aim at grabbing receivers’ attention. This further provides evidence in favour of the hypothesis that the use of roughness in alarm signalling is not an epiphenomenon of vocal production. Instead, the selection of communicative features depends on their propensity to enhance the detectability of vocal utterances and to induce adapted behavioural responses. In this view, the use of roughness in alarm signals may reflect an adaptation of communication to the receiver’s auditory sampling constraints to hijack her brain, enhance her perception of incoming danger and manipulate her reactions from a distance to ultimately promote survival.

## Data Availability

This article has no additional data.
